# Upper Blepharoplasty Under Anticoagulant or Antiplatelet Therapy: Is Safety Still a Concern?

**DOI:** 10.1007/s00266-026-05944-7

**Published:** 2026-06-03

**Authors:** Juan A. Viscardi, Salvatore Giordano

**Affiliations:** Department of Plastic and General Surgery, Turku University Hospital and The University of Turku, Turku, Finland

**Keywords:** Anticoagulation, Dermatochalasis, Blepharoplasty, Antiplatelet therapy, Bleeding-related complications, Upper eyelid

## Abstract

**Objective:**

Dermatochalasis is defined as a skin excess in the upper or lower eyelid which may be associated with orbital fat prolapse, lacrimal gland prolapse, and involutional blepharoptosis. Upper blepharoplasty is the gold-standard procedure for correcting dermatochalasis and is the third most common cosmetic surgery performed in the United States. However, it is frequently performed on elderly patients, who are often ongoing anticoagulant and/or antiplatelet therapy. Due to the associated elevated risk of bleeding, it remains unclear whether upper blepharoplasty can be safely performed in this patient population. This study aimed to investigate the safety of upper blepharoplasty in patients receiving antithrombotic therapy.

**Material and Methods:**

A retrospective comparative study was conducted on 392 consecutive patients who underwent upper blepharoplasty for dermatochalasis at a single academic center over a three-year period. Patients with a history of prior eyelid or orbital surgery were excluded. The cohort was stratified into two groups: a study group of 100 patients on anticoagulant/antiplatelet therapy (including aspirin, warfarin, and apixaban) and a control group of 292 patients not on such therapy. Postoperative complications, clinical outcomes, and satisfaction scores (from both patients and surgeons) were compared between the groups.

**Results:**

Baseline characteristics differed significantly between groups, with patients on anticoagulant/antiplatelet therapy being older and having higher comorbidity rates. Operative time, estimated blood loss, time to return to work, and follow-up duration were comparable. The overall complication rate was not significantly different between the antithrombotic and control groups (6.0% vs. 3.5%, *p*=0.380). Notably, no major bleeding events occurred. The incidence of ecchymosis requiring extended observation was low and not significantly different (1.0% vs. 0.0%, *p*=0.573). Patient-reported and surgeon-assessed satisfaction scores were high and similar between groups. Reoperation rates were also equivalent. On multivariable logistic regression analysis, no variable was identified as a significant independent predictor of complications; however, hypertension demonstrated the highest odds ratio (OR 2.9, 95% CI 0.29-28.56, *p*=0.370).

**Conclusions:**

Upper blepharoplasty appears to be safely performed in carefully selected patients receiving anticoagulant or antiplatelet therapy. In this retrospective cohort, continuation of antithrombotic therapy was not associated with an increased rate of detected complications compared with controls. These findings support the feasibility of maintaining therapy in appropriately selected cases, although larger prospective studies are required to confirm these observations.

**Level of Evidence III:**

This journal requires that authors assign a level of evidence to each article. For a full description of these Evidence-Based Medicine ratings, please refer to the Table of Contents or the online Instructions to Authors www.springer.com/00266.

## Introduction

As the gold standard for treating dermatochalasis, blepharoplasty is one of the most common plastic surgery treatments in the US, with over 200,000 procedures performed each year [1].

Dermatochalasis is defined as an excess of skin affecting either the upper or lower eyelids and the resulting visual field limitation has been associated with persistent tension-type headaches due to restricted visual input [2–4]. According to recent data from the Aesthetic Society’s 2023 Aesthetic Plastic Surgery Statistics Report, aesthetic operations increased by 14% overall in 2022, with blepharoplasty showing a significant 35% increase in incidence from 2019 [5]. Functional advantages of upper blepharoplasty include improved quality of life for headache patients due to a reduction in occipitofrontal muscle overactivity and an expansion of the visual field by lowering peripheral visual blockage [6–8]. A recent meta-analysis who reviewed exclusively randomized controlled trials by Todorov et al. reported a significant reduction in dry eye symptoms following upper blepharoplasty, corroborating earlier evidence that surgical correction of the eyelids can enhance eyelid function and decrease ocular surface exposure, thereby improving dry eye conditions [9]. Overall, upper blepharoplasty offers patients comprehensive advantages for personal well-being by addressing functional limitations as well as improving quality of life, self-esteem, and appearance satisfaction [1, 2, 4, 7, 10–14].

**Fig. 1:**
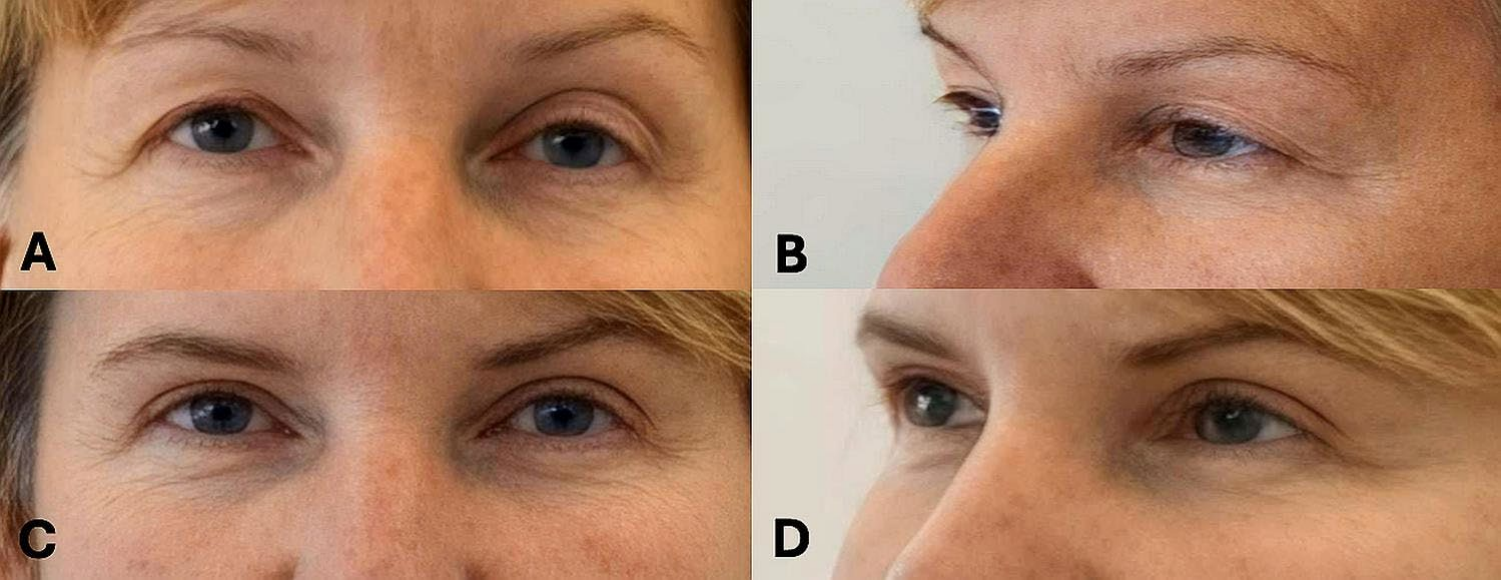
Preoperative view of a 59-year-old female patient (**A**, **B**), and 6 months after skin-only blepharoplasty (**C**, **D**)

Up to 14% of patients can experience at least one complication, these include symptomatic dryness, asymmetry, periorbital hematoma, chemosis, surgical site infections, suture granulomas, lagophthalmos and post-operative ptosis as well as lagophthalmos and hollowed appearance due to excessive fat removal [15–21]. Visual loss due to ischemia or retrobulbar hemorrhage is the most feared complication. This rare complication requires immediate surgical surgery for decompression, with estimations showing an incidence of about 0.04% [16, 17, 20, 22–25]. There is still uncertainty regarding the safety of blepharoplasty in patients on anticoagulant or antiplatelet therapy, as well as if perioperative treatment suspension is required (Fig. 1).

Anticoagulant and antiplatelet therapies are increasingly prescribed in the aging population, particularly among patients with cardiovascular comorbidities [26–28]. As upper blepharoplasty is frequently performed in older individuals, surgeons are often confronted with the perioperative dilemma of whether antithrombotic therapy should be continued or temporarily discontinued. Although treatment interruption may theoretically reduce bleeding risk, it may simultaneously expose patients to potentially serious thromboembolic complications [29–32]. While accumulating evidence from dermatologic and facial procedures suggests that continuation of antithrombotic therapy may be safe in selected settings, data specifically evaluating upper blepharoplasty remain limited [33–36].

The American Board of Plastic Surgery continuous certification tracer data, which examined more than 3,000 cases from 2005 to 2020, indicates that blepharoplasty is most frequently performed on middle-aged patients, with a mean age of 56 years old. Of these, 2% had a history of anticoagulant/antiplatelet treatment, and 32% had concurrent comorbidities [21].

In Finland, when dermatochalasis leads to functional impairment, blepharoplasty is provided within the public health care system. The aim of this study is to evaluate the safety of upper blepharoplasty in a reconstructive setting among patients receiving continuous anticoagulant or antiplatelet therapy, such as aspirin, warfarin, or apixaban. We hypothesized that upper blepharoplasty performed without interruption of antithrombotic therapy would not be associated with a clinically meaningful increase in postoperative complications.

## Material and Methods

### Study Design

This retrospective cohort study was conducted at a single academic center on patients who underwent upper blepharoplasty over a three-year period. The study was approved by the local institutional review board and was conducted in accordance with the principles of the Declaration of Helsinki. In accordance with national guidelines, formal ethical committee approval was waived for this retrospective chart review study. The study population was identified using the hospital’s computerized medical records system.

### Study Population

Consecutive patients who underwent bilateral upper blepharoplasty for dermatochalasis during the study period were initially identified (*n*=404). The diagnosis of dermatochalasis was based on functional indications, including a margin reflex distance of less than 2 mm, excess upper eyelid skin causing visual obstruction or ocular irritation (e.g., skin touching the eyelashes or cornea), or skin folding leading to inflammation. Patients with a history of prior orbital or eyelid surgery were excluded (*n*=12), resulting in a final cohort of 392 patients.

Patients were stratified into two groups based on their medication history: a study group (*n*=100) consisting of patients on ongoing anticoagulant (warfarin, apixaban) or antiplatelet (aspirin) therapy, and a control group (*n*=292) consisting of patients not on any such therapy.

### Data Collection

Data were extracted through a comprehensive review of electronic medical records. Collected variables included: Patient demographics, comorbidities, and use of medications known to increase bleeding risk.

Surgical technique, equipment used, type and dosage of local anesthetic, and operative time.

Duration of sick leave, length of follow-up, complications (e.g., hematoma, infection, wound dehiscence), and rates of reoperation.

Postoperative complications, defined as wound dehiscence, infection, erythema, or unacceptable scarring, served as the primary outcome measure. Secondary outcomes involved the rate of surgical revisions and the presence of functional upper eyelid anomalies, including persistent lagophthalmos, ptosis, and xerophthalmia. Furthermore, both patient and surgeon satisfaction with the final aesthetic outcome were quantitatively assessed at the last postoperative follow-up visit using a non-validated 10-point visual analogue scale (VAS), routinely employed in our clinical practice, ranging from 0 (not satisfied) to 10 (completely satisfied). The assessment was performed by the operating surgeon during the standard postoperative evaluation.

### Statistical Analysis

For this study, descriptive statistics are expressed as means with standard deviations (SD) for continuous variables and as numbers with percentages (%) for categorical variables. Data distribution was evaluated for normality using histograms, skewness/kurtosis values, and the Kolmogorov-Smirnov test. Inter-group differences for continuous variables were analyzed with the independent samples Student’s *t*-test (normal distribution) or the Mann-Whitney U test (non-normal distribution). Associations between categorical variables were examined using Pearson’s chi-square test, with Fisher’s exact test employed for expected cell counts below five. A multivariable logistic regression analysis was conducted to control for potential confounding factors and to identify independent predictors of postoperative complications. Results are presented as adjusted odds ratios (aORs) with 95% confidence intervals (CIs). Model fit was assessed using the Hosmer–Lemeshow test, which indicated good fit (*p* = 0.448). A post hoc power analysis was performed to estimate the statistical power to detect the observed difference in complication rates between groups, using a two-sided alpha level of 0.05. The threshold for statistical significance was set at *p* < 0.05. All analyses were performed using IBM SPSS Statistics, Version 30.0 (IBM Corp., Armonk, NY, USA).

## Results

Patients in the study group were significantly older than those in the control group (72.5 ± 8.8 years vs. 65.1 ± 9.9 years, *p* < 0.001) and had a higher prevalence of hypertension (84 [84.0%] vs. 128 [44.4%], *p* < 0.001), diabetes (33 [33.0%] vs. 26 [9.1%], *p* < 0.001), and hypercholesterolemia (66 [66.0%] vs. 75 [26.6%], *p* < 0.001). A significant difference was also observed in the prevalence of preoperative ptosis (10 [10.0%] vs. 8 [2.8%], *p* = 0.010). No other significant demographic differences were observed between the groups (Table 1).

**Table 1:** Demographics of patients at time of study

	Study group (*n* = 100)	Control group (*n* = 292)	*p*-value
Age (mean ± SD)	72.5 ± 8.8	65.1 ± 9.9	0.001
BMI	27.2 ± 6.5	27.6 ± 6.4	0.652
Any comorbidity	81 (81.0%)	158 (55.4%)	<0.001
HTA	84 (84.0%)	128 (44.4%)	<0.001
Diabetes	33 (33.0%)	26 (9.1%)	<0.001
Hypercholesterolemia	66 (66.0%)	75 (26.6%)	<0.001
Lung disease	19 (19.0%)	36 (12.5%)	0.108
Depression	14 (14.0%)	29 (10.1%)	0.273
Smokers	15 (15.0%)	44 (15.3%)	1.000
Warfarin	30 (30.0%)	0 (0.0%)	
Aspirin	66 (66.0%)	0 (0.0%)	
Apixaban	10 (10.0%)	0 (0.0%)	
Ptosis	10 (10.0%)	8 (2.8%)	0.010
Asymmetry	34 (34.0%)	82 (28.6%)	0.308

No significant differences were observed between the groups in operative time, estimated intraoperative blood loss, or duration of sick leave. The overall mean follow-up duration was 4.5 ± 15.6 months, with no significant difference between the study and control groups (Table 2).

**Table 2:** Comparison of perioperative parameters in the two groups of patients

	Study group (*n* = 100)	Control group (*n* = 292)	*p*-value
Operative time (min, mean ± SD)	65.2 ± 19.0	64.4 ± 17.2	0.845
Estimated blood loss (ml, mean ± SD)	8.2 ± 6.5	6.0 ± 3.8	0.176
Return to work (days, mean ± SD) *	8.2 ± 4.3	8.8 ± 3.6	0.756
Follow-up (months, mean ± SD)	3.66 ± 9.3	4.8 ± 17.2	0.541

^*^For patients not retired (6 vs. 88 patients)

The overall complication rate was not significantly different between the study and control groups (6.0% vs. 3.5%, *p*=0.380). This included specific complications such as infection, ecchymosis, postoperative ptosis, and levator damage, none of which differed significantly. The reoperation rate was comparable between groups (8.0% vs. 8.7%, *p*=0.999). Finally, both patient-reported and surgeon-assessed satisfaction scores showed no statistically significant differences (Table 3).

**Table 3:** Postoperative complications and satisfaction

	Study group (*n* = 100)	Control group (*n* =292)	*p*-value
Complications	6 (6.0%)	10 (3.5%)	0.380
Any infection	0 (0.0%)	4 (1.4%)	0.576
Ecchymosis (requiring any observation)	0 (0.0%)	3 (1.0%)	0.573
Postoperative ptosis	0 (0.0%)	2 (0.7%)	1.000
Levator damage	0 (0.0%)	0 (0.0%)	1.000
Re-operation	8 (8.0%)	25 (8.7%)	0.841
Patients’ satisfaction (0–10, mean ± SD)	8.1 ± 2.6	7.6 ± 2.6	0.411
Surgeon’s satisfaction (0–10, mean ± SD)	8.6 ± 2.2	7.8 ± 2.5	0.209

On multivariable analysis, no factor was independently associated with an increased risk of complications. Hypertension yielded the highest, albeit non-significant, odds ratio (OR 2.9, 95% CI 0.29–28.56, *p*=0.370) (Table 4). The post hoc power analysis demonstrated a power of approximately 19% to detect the observed difference in complication rates between groups.

**Table 4:** Multivariable logistic regression was used to assess independent risk factors for complications, with adjusted odds ratios provided

	Odds ratio	95% CI	*p*-value
HTA	2.86	0.29–28.56	0.370
Diabetes	1.06	0.06–20.27	0.968
Operative time	1.03	0.98–1.08	0.239
Age	1.02	0.91–1.14	0.786
Estimated blood loss	1.00	0.81–1.23	0.996
Smoking	0.99	0.12–8.19	0.994
BMI	0.88	0.69–1.12	0.304
Depression	0.65	0.04–11.97	0.774
Hypercholesterolemia	0.62	0.09–4.44	0.631

*CI* confidence interval

## Discussion

The current study aimed to assess the safety and aesthetic outcomes of upper blepharoplasty in patients on anticoagulant or antiplatelet therapy, compared to a control group, in a reconstructive setting at a university hospital. Upper blepharoplasty is the gold-standard procedure for correcting dermatochalasis, which improves the functional, aesthetic, and psychological impairments associated with this condition [2, 3, 12, 15, 16, 37–40].

Our findings highlight significant baseline differences between the study and control groups. Most notably, patients in the anticoagulant/antiplatelet group were significantly older and had a higher burden of comorbidities. This disparity reflects the typical clinical profile of patients requiring such therapy, who often present with multiple underlying conditions such as hypertension, diabetes, and hyperlipidemia [41–44]. Notably, despite a substantially higher comorbidity burden and older age in the study cohort, postoperative outcomes remained comparable, supporting the real-world applicability of these findings. No significant difference was found in the prevalence of smoking or depression between the groups. This finding corroborates the distinct baseline health profile associated with anticoagulant/antiplatelet therapy, which is typically prescribed for older patients with a higher incidence of cardiovascular and metabolic comorbidities, rather than behavioural health conditions.

To mitigate risks in elderly patients, surgeons may prefer less invasive approaches for dermatochalasis correction to limit complications associated with advanced age and comorbidities, such as increased bleeding. However, as previously reported by Viscardi et al. in the same Finnish Caucasian study population, no significant differences in surgical outcomes were observed when comparing blepharoplasty techniques performed with or without orbicularis oculi muscle resection or preaponeurotic fat pad removal [45].

No statistically significant differences were observed between the study and control groups in operative time, duration of sick leave, or estimated intraoperative blood loss. Operative time and blood loss are inherently related to surgical complexity, as techniques more invasive than simple skin excision naturally require more time. The absence of statistical differences in these parameters indicates that the continuation of anticoagulant/antiplatelet therapy does not necessitate technical modifications during surgery, thereby supporting the procedure’s safety and reproducibility in this patient population. Our findings are particularly relevant as they challenge prior recommendations advocating for the discontinuation or tailoring of antithrombotic therapy before oculoplastic surgery. Instead, we demonstrate that these medications can be safely maintained without increasing perioperative risk [29–32].

The findings of this study align with an expanding corpus of literature on cutaneous surgery, including facial surgeries, which collectively emphasize that the continuation of antithrombotic medication is both viable and secure. These studies emphasize that the risk of perioperative bleeding from the continuation of antithrombotic agents is typically minimal in both severity and clinical relevance, and that these risks are significantly outweighed by the significantly larger implications of thromboembolic complications, which may result in life-threatening or fatal events [33–36].

Minor complications occurred in 6.0% of patients in the study group and 3.5% in the control group; no major complications were recorded in either cohort. This difference in complication rates was not statistically significant. Similarly, the reoperation rate was comparable between the study (8.0%) and control (8.7%) groups, a difference which also lacked statistical significance. Particularly, patient and surgeon satisfaction, evaluated via a visual analogue scale, showed no significant intergroup differences.

These findings together indicate that despite ongoing anticoagulant or antiplatelet therapy, procedural safety profiles remained comparable. The consistent surgical strategy across both groups successfully achieved equivalent satisfaction outcomes. Furthermore, the specific technical variations employed, for example, with or without orbicularis oculi muscle resection and with or without preaponeurotic or nasal fat pad removal, all appeared to be safe for patients on antithrombotic therapy, with no associated increase in complications relative to controls.

This study has several limitations inherent to its design and setting. Notably, it was not designed as an equivalence or non-inferiority analysis; therefore, the absence of statistically significant differences should be interpreted as a lack of detected increased risk rather than as evidence of equivalence between groups. The low post hoc power indicates that the study may have been underpowered to detect small differences in rare complications. Firstly, its retrospective, non-randomized nature introduces potential for selection bias and unmeasured confounding. Although we controlled for key variables in a multivariable analysis, the baseline differences in age and comorbidity burden between groups suggest that unaccounted factors may have influenced both the decision to continue anticoagulant/antiplatelet therapy and the surgical outcomes.

Secondly, all procedures were performed within a single academic, reconstructive surgery unit. This setting, which focuses on functional correction rather than purely aesthetic outcomes, may limit the generalizability of our findings to private practice or exclusively aesthetic surgery settings where patient demographics and surgical techniques might differ.

Thirdly, the assessment of patient and surgeon satisfaction, while valuable, was conducted using a non-validated instrument (the VAS scale) administered by the operating surgeon. This method introduces the potential for acquiescence bias and may not fully capture the nuances of postoperative satisfaction.

Finally, the sample size, particularly in the antithrombotic group (*n* = 100), may have limited the ability to detect small differences in rare complications; therefore, a type II error cannot be excluded. Accordingly, the present findings should be interpreted as indicating no detected increase in risk rather than as evidence of true equivalence between groups.

To our knowledge, this represents one of the largest comparative studies evaluating the safety and outcomes of upper blepharoplasty in patients ongoing anticoagulant or antiplatelet therapy within a reconstructive surgery setting. Although the sample size in the study group may have limited the power to detect very small differences in complication and reoperation rates, our findings robustly demonstrate no significant increase in risk compared to matched controls. Based on these results, we conclude that continuation of anticoagulant/antiplatelet therapy is a safe and viable option for most patients undergoing this procedure. Given the increasing use of antithrombotic therapy, large-scale prospective multicenter studies are needed to definitively confirm these findings and to clarify whether outcomes differ between specific anticoagulant/antiplatelet agents. Future prospective multicenter studies with larger cohorts are warranted to further validate these results and refine surgical recommendations for this patient population.

## Conclusion

Continuation of anticoagulant or antiplatelet therapy during upper blepharoplasty was not associated with an increased rate of observed complications in this retrospective cohort. These findings suggest that, in appropriately selected patients undergoing eyelid surgery, continuation of antithrombotic therapy may be a reasonable option without an apparent increase in observed complications. Given the retrospective design and limited ability to detect small differences in rare events, individualized clinical judgment remains essential, and further prospective studies are warranted to confirm these findings.
